# Nonconserved miR‐608 suppresses prostate cancer progression through RAC2/PAK4/LIMK1 and BCL2L1/caspase‐3 pathways by targeting the 3′‐UTRs of RAC2/BCL2L1 and the coding region of PAK4

**DOI:** 10.1002/cam4.2455

**Published:** 2019-08-07

**Authors:** Xu Zhang, Jiajie Fang, Shiming Chen, Weiyu Wang, Shuai Meng, Ben Liu

**Affiliations:** ^1^ Department of Urology the First Affiliated Hospital, Zhejiang University School of Medicine Hangzhou China; ^2^ Department of Urology Zhejiang Provincial People's Hospital Hangzhou China

**Keywords:** BCL2L1, G2/M arrest; RAC2, microRNA‐608, PAK4, prostate cancer

## Abstract

The aim of this study is to investigate the functions and mechanisms of miR‐608 in prostate cancer (PCa). CISH and qRT‐PCR analysis demonstrated that miR‐608 was low expressed in PCa tissues and cells, which was partly attributed to the methylation of CpG island adjacent to the transcription start site (TSS) of miR‐608 gene. Intracellular miR‐608 overexpression inhibited in vivo PCa tumor growth, and suppressed PCa cell proliferation, G2/M transition, and migration in vitro, which was independent of EMT‐associated mechanisms. Then RAC2, a GTPase previously deemed hematopoiesis‐specific but now discovered to exist and play important roles in PCa, was verified by western blot and dual‐luciferase reporter assays to mediate the effects of miR‐608 through RAC2/PAK4/LIMK1/cofilin pathway. MiR‐608 also promoted the apoptosis of PCa cells through BCL2L1/caspase‐3 pathway by targeting the 3′‐UTR of BCL2L1. Moreover, PAK4, the downstream effector of RAC2, was found to be targeted by miR‐608 at the mRNA coding sequence (CDS) instead of the canonical 3′‐UTR. Knocking down RAC2, PAK4, or BCL2L1 with siRNAs reproduced the antiproliferative, mitosis‐obstructive, antimigratory and proapoptotic effects of miR‐608 in PCa cells, which could be attenuated by downregulating miR‐608. In conclusion, miR‐608 suppresses PCa progression, and its activation provides a new therapeutic option for PCa.

## 1 INTRODUCTION

Recent statistics revealed that prostate cancer (PCa) is the second prevalent cancer and incurs the fifth most deaths in men globally.^1^ Although the combination of modern surgery, radiotherapy, and androgen‐deprivation therapy could remarkably enhance the prognoses of patients with PCa, the progression from androgen‐sensitive prostate cancer to castration‐resistant prostate cancer (CRPC) remains formidable and poses considerable challenges to PCa treatment.

Among the presently developing regimens for PCa, RNAi therapies which are based on the discovery of RNA interference and microRNA (miRNA) have shown promising prospects.^2^, ^3^ MiRNA is a 21‐23 nucleotide‐length endogenous noncoding RNA, which usually functions by targeting mRNA 3′‐untranslated regions (3′‐UTR). In this way, it causes the degradation or transcriptive silencing of mRNAs, and therefore regulates multiple biological activities, such as metabolism, development, differentiation, immune response, oncogenesis, and so forth.^4^, ^5^ On the other hand, miRNA could also downregulate its target proteins by interacting with the coding sequences (CDS) of their mRNAs instead of 3′‐UTRs, in a way similar to artificially synthesized small interfering RNA (siRNA).^6^, ^7^

MiR‐608 is a nonconserved miRNA derived from an intron of human SEMA4G (semaphorin 4G) gene located at chromosome 10q24.31.^8^ It has been implicated in many malignancies, such as pancreatic cancer, bladder cancer, ovarian cancer, and lung adenocarcinoma, where miR‐608 inhibits their proliferation and progression.^9^, ^10^, ^11^, ^12^ However, the particular functions of miR‐608 in PCa have not been investigated. In this research, for the first time, we revealed that miR‐608 was low expressed in PCa cell lines and tissues, and overexpression of miR‐608 suppressed the proliferation, induced G2/M arrest, promoted the apoptosis, and inhibited the migration of PCa cells. Also, RAC2, a GTPase previously considered to be specifically expressed in hematopoietic cells, was discovered to exist in PCa and exert significant effects through RAC2/PAK4/LIMK1/cofilin signaling pathway. We confirmed that miR‐608 targeted RAC2 and antiapoptotic factor BCL2L1 in PCa by binding to their 3′‐UTRs. Moreover, PAK4, a downstream effector of RAC2, was found to be targeted by miR‐608 at the CDS of its mRNA instead of the canonical 3′‐UTR. These findings revealed the novel underlying mechanisms of miR‐608 in PCa tumor suppression.

## 2 MATERIALS AND METHODS

### 2.1 Cell culture

DU145 and PC3 PCa cell lines, RWPE‐1 prostatic epithelial cell line and HEK 293T cell line were purchased from Stem Cell Bank, Chinese Academy of Sciences. PCa cell lines and HEK 293T cell line were respectively cultured in minimal essential medium (MEM) and Dulbecco's modified Eagle's medium (DMEM), which were improved with 10% fetal bovine serum (FBS, Biological Industries), while RWPE‐1 cell line was cultured in keratinocyte serum free medium (K‐SFM, Gibco). All cells were cultured in 37°C thermostatic atmospheres with 5% CO_2_.

### 2.2 Transient transfection of RNA oligonucleotides

MicroRNA mimics, siRNA duplexes, and microRNA inhibitors were produced by GenePharma. MicroRNA‐608 mimic was employed for the gain‐of‐function studies and single‐stranded microRNA‐608 inhibitor was utilized for the rescue experiments. SiRNAs targeting RAC2/PAK4/BCL2L1 mRNA were designed by GenePharma. In order to diminish off‐target effects, two siRNAs were designed and synthesized, and mixed as a siRNA pool for each target. Negative control microRNA duplex was designated NC and was heterologous to all human genome sequences. All the RNA oligo sequences are shown in Table 1. Lipofectamine 2000 (Invitrogen) was employed for transient transfection.

**Table 1:** Sequences of oligonucleotides used in the research

Name^a^	Sequences (5′‐3′)^b^
miR‐608 mimic (sense)	AGGGGUGGUGUUGGGACAGCUCCGU
NC (sense)	UUCUCCGAACGUGUCACGU
miR‐608 inhibitor	ACGGAGCUGUCCCAACACCACCCCU
inhibitor NC	CAGUACUUUUGUGUAGUACAA
miR‐608 F	AGGGGTGGTGTTGGGACAGCTCCG
miR‐608 probe^c^	ACGGAGCTGTCCCAACACCACCCCT
RAC2 F	CAACGCCTTTCCCGGAGAG
RAC2 R	TCCGTCTGTGGATAGGAGAGC
PAK4 F	ATCTGGTCGCTGGGGATAATG
PAK4 R	CAGGTTGTCCCGAATCATCTTC
BCL2L1 F	GACTGAATCGGAGATGGAGACC
BCL2L1 R	GCAGTTCAAACTCGTCGCCT
GAPDH F	CTGGGCTACACTGAGCACC
GAPDH R	AAGTGGTCGTTGAGGGCAATG
RAC2 3′‐UTR Wt F	CATCTACCCGTTCACTCCAGTC**CCACCCC**ACGCCTGACTCCCCTCTGGAAG
RAC2 3′‐UTR Wt R	TCGACTTCCAGAGGGGAGTCAGGCGT**GGGGTGG**GACTGGAGTGAACGGGTAGATGAGCT
RAC2 3′‐UTR Mut F	CATCTACCCGTTCACTCCAGTCGGTGGGGACGCCTGACTCCCCTCTGGAAG
RAC2 3′‐UTR Mut R	TCGACTTCCAGAGGGGAGTCAGGCGTCCCCACCGACTGGAGTGAACGGGTAGATGAGCT
PAK4 3′‐UTR Wt‐1 F	CGTCAGCGCAGCCCCAGCCCGC**CCACCCCT**GCCTCGAGTTAGTTTTACAAG
PAK4 3′‐UTR Wt‐1 R	TCGACTTGTAAAACTAACTCGAGGC**AGGGGTGG**GCGGGCTGGGGCTGCGCTGACGAGCT
PAK4 3′‐UTR Mut‐1 F	CGTCAGCGCAGCCCCAGCCCGCGGTGGGGAGCCTCGAGTTAGTTTTACAAG
PAK4 3′‐UTR Mut‐1 R	TCGACTTGTAAAACTAACTCGAGGCTCCCCACCGCGGGCTGGGGCTGCGCTGACGAGCT
PAK4 3′‐UTR Wt‐2 F	CTGTGTGTGTGCAAAGGTCCAG**CCACCCC**GTCCTCCAGCCTGCAAGGGGTG
PAK4 3′‐UTR Wt‐2 R	TCGACACCCCTTGCAGGCTGGAGGAC**GGGGTGG**CTGGACCTTTGCACACACACAGAGCT
PAK4 3′‐UTR Mut‐2 F	CTGTGTGTGTGCAAAGGTCCAGGGTGGGGGTCCTCCAGCCTGCAAGGGGTG
PAK4 3′‐UTR Mut‐2 R	TCGACACCCCTTGCAGGCTGGAGGACCCCCACCCTGGACCTTTGCACACACACAGAGCT
PAK4 CDS Wt F	CGAGCCCCCCTACTTCAACGAG**CCACCCCT**CAAAGCCATGAAGATGATTCGGGACAACCTG**CCACCCC**GACTGAAGAACCTGCACAAGGG
PAK4 CDS Wt R	TCGACCCTTGTGCAGGTTCTTCAGTC**GGGGTGG**CAGGTTGTCCCGAATCATCTTCATGGCTTTG**AGGGGTGG**CTCGTTGAAGTAGGGGGGCTCGAGCT
PAK4 CDS Mut F	CGAGCCCCCCTACTTCAACGAGGGTGGGGACAAAGCCATGAAGATGATTCGGGACAACCTGGGTGGGGGACTGAAGAACCTGCACAAGGG
PAK4 CDS Mut R	TCGACCCTTGTGCAGGTTCTTCAGTCCCCCACCCAGGTTGTCCCGAATCATCTTCATGGCTTTGTCCCCACCCTCGTTGAAGTAGGGGGGCTCGAGCT
BCL2L1 3′‐UTR Wt‐1 F	CCTGACCATCCACTCTACCCTC**CCACCCC**CTTCTCTGCTCCACCACATCCG
BCL2L1 3′‐UTR Wt‐1 R	TCGACGGATGTGGTGGAGCAGAGAAG**GGGGTGG**GAGGGTAGAGTGGATGGTCAGGAGCT
BCL2L1 3′‐UTR Mut‐1 F	CCTGACCATCCACTCTACCCTCGGTGGGGCTTCTCTGCTCCACCACATCCG
BCL2L1 3′‐UTR Mut‐1 R	TCGACGGATGTGGTGGAGCAGAGAAGCCCCACCGAGGGTAGAGTGGATGGTCAGGAGCT
BCL2L1 3′‐UTR Wt‐2 F	CCATCTGCCCCTCCCCCAACCC**CCACCCC**ACACTTGTTCCAGCTCTTTGAG
BCL2L1 3′‐UTR Wt‐2 R	TCGACTCAAAGAGCTGGAACAAGTGT**GGGGTGG**GGGTTGGGGGAGGGGCAGATGGAGCT
BCL2L1 3′‐UTR Mut‐2 F	CCATCTGCCCCTCCCCCAACCCGGTGGGGACACTTGTTCCAGCTCTTTGAG
BCL2L1 3′‐UTR Mut‐2 R	TCGACTCAAAGAGCTGGAACAAGTGTCCCCACCGGGTTGGGGGAGGGGCAGATGGAGCT
RAC2 siRNA 1 (sense)	CCAAGUGGUUCCCAGAAGU
RAC2 siRNA 2 (sense)	CCACCUAGAUGGGUCUGAU
PAK4 siRNA 1 (sense)	GCUCCUACCUGGACAACUU
PAK4 siRNA 2 (sense)	CAGCAAAGGUGCCAAAGAU
BCL2L1 siRNA 1 (sense)	CAGCAUAUCAGAGCUUUGA
BCL2L1 siRNA 2 (sense)	GGAACUCUAUGGGAACAAU

a F: forward primer or sense oligomer; R: reverse primer or antisense oligomer.

b Wild‐type binding sites are in bold and mutant‐type binding sites are underlined.

c 5′‐DIG and 3′‐DIG double labeled.

### 2.3 Chromogenic in situ hybridization (CISH)

A digoxigenin (DIG) double‐labeled miR‐608 probe (Servicebio) was used for miR‐608 detection in PCa tissue microarray (TMA) (IWLT‐N‐91P61, Iwill Biological Technology) which consisted of 32 cases of PCa samples and paired peritumoral tissues. After deparaffinization, PCa TMA was treated with proteinase K to expose RNA antigens. Subsequently, hybridization was performed on TMA with 10 ng miR‐608 probes at 4°C for 2 days. Finally miR‐608 chromogenesis was achieved by incubating TMA with HRP‐Conjugated IgG Fraction Anti‐DIG (Jackson ImmunoResearch) and DAB development. MiR‐608 positivity was semiquantitatively analyzed based on the proportion and intensity of the miR‐608‐positive cells.

### 2.4 DNA methylation analysis

To investigate the methylation state of CpG‐islands close to miR‐608 transcription start site (TSS) in PCa cells, bisulfite sequencing PCR (BSP) was utilized. Forward primer 5′‐TATTTTATTTTTTAAGTTGGGTTAGG‐3′ and reverse primer 5′‐CCCTCCAACATCCTAAACAATC‐3′ were adopted to amplify the identified CpG‐island DNA sequence. Amplified PCR products were isolated, purified, and inserted into pUC18‐T vectors constructed by Sangon. Correct insertion of the CpG‐island sequence was verified by blue‐white screen of *E.coli* DH5α competent cells (Sangon), and 10 positive single colonies were sequenced by BSP (Sangon).

PC3 cells were treated with 5 μmol/L 5‐aza‐2′‐deoxycytidine (Sigma Aldrich) for 72 hours. Later RNA of PC3 cells was extracted and miR‐608 was quantified as per the section qRT‐PCR.

### 2.5 Cell viability assay

PCa cells were seeded in 96‐well plates which had 6 × 10^3^ cells in each well and cultured overnight. Then miR‐608 mimic/RAC2 siRNA/PAK4 siRNA of different concentrations ranging from 0 nmol/L to 75 nmol/L were transfected into PCa cells. Forty‐eight or 72 hours after transfection, culture medium was replaced with Cell Counting Kit 8 (CCK8, Dojindo) reagent dissolved in nine volumes of complete MEM. After 1‐hour incubation at 37°C, the absorbance at 450 nm wavelength of each well was measured with Elx800 absorbance reader (BioTek Instruments). The relative viability was presented as the ratio of mean absorbance of each group to that of mock group.

### 2.6 Colony formation assay

MiR‐608 mimic/RAC2 siRNA/PAK4 siRNA‐transfected PCa cells were harvested 48 hours after transfection and reseeded in 6‐well plates which had 500 cells in each well. Again cells were cultured under normal conditions. After 10 days, colonies were visualized by 100% methanol fixing and 0.1% crystal violet staining (Solarbio). Colonies over 1 mm in diameter were tallied.

### 2.7 Subcutaneous tumorigenesis assay

BALB/c nude mice (male, 4 weeks old) were supplied by Laboratory Animal Research Center of Zhejiang Chinese Medical University (Hangzhou, China). Each mouse was subcutaneously injected at left flank with 2 × 10^6^ PC3 cells suspended in 200 μL PBS. When xenograft tumors reached about 5 mm in diameter, each mouse was intratumorally injected with 30 μg miR‐608 mimic or NC which were encapsulated in Lipofectamine 2000. Injections were carried out every 4 days for seven times. Every 4 days two perpendicular diameters of each xenograft tumor were measured, and formula V = π/6 × length × width^2^ was applied for tumor volume calculation. The Institutional Animal Care and Use Committee of Zhejiang Chinese Medical University approved the use of animals in this study in compliance with relevant experiment guidelines, and the ethical approval code was 2018010802.

### 2.8 Flow cytometry cell cycle assay

PCa cells transfected with miR‐608 mimic/RAC2 siRNA/PAK4 siRNA were collected 48 hours after transfection and fixed at −20°C overnight in 75% ethanol. Later cells were gathered and treated with propidium iodide (Liankebio). FACSCantoⅡ flow cytometry (BD) and ModFit 4.0 software were used for cell cycle analysis.

### 2.9 Flow cytometry apoptosis and active caspase‐3 assay

Seventy‐two hours after miR‐608 mimic/BCL2L1 siRNA transfection, all PCa cells (including cells in medium) were collected and treated with FITC‐Annexin and propidium iodide (Liankebio) or CaspGLOW Fluorescein Active Caspase‐3 Staining Kit (Invitrogen). FACSCantoⅡ flow cytometry (BD) and FlowJo 10.0 software were used for apoptosis and active caspase‐3 analyses.

### 2.10 Transwell migration assay

Twenty‐four hours after miR‐608 mimic/RAC2 siRNA/PAK4 siRNA transfection, PCa cells were collected and suspended in serum‐free MEM, and 10^5^ cells were reseeded in Millicell 24‐Well Hanging Inserts (Millipore). The hanging inserts containing PCa cells were mounted in 24‐well plates with 700 μL complete MEM per well and placed back to standard culture environment. Twenty‐four hours later cells in upper chambers were discarded and cells in lower chambers were visualized by 100% methanol fixing and 0.1% crystal violet staining (Solarbio). Finally, the membranes on which cells attached were mounted on slides, and then observed microscopically at a magnification of 200×.

### 2.11 Quantitative real‐time PCR (qRT‐PCR)

RNAiso Plus was employed for RNA extraction 48 hours after transfection. PrimeScript RT Master Mix was adopted for reverse transcription of mRNA, and miRNA was converted into tailed cDNA with Mir‐X MiRNA First Strand Synthesis Kit. TB Green Premix Ex Taq Ⅱ was used for cDNA amplification, and quantification was accomplished by CFX96 system (Bio‐Rad). All PCR reagents were purchased from Takara. Table 1 shows all primer sequences. Results were analyzed by 2^−△△Ct^ method.

### 2.12 Western blot analysis

Forty‐eight hours after transfection, PCa cells were collected and lysed on ice for 30 minutes with RIPA lysis buffer (Servicebio) which contained protease and phosphatase inhibitor (Pierce Mini Tablets, Thermo Scientific). BCA assay was employed to determine protein amounts. Protein samples were electrophoresed and separated in 4%‐20% polyacrylamide gels (GenScript) and electrotransferred to polyvinylidene fluoride (PVDF) membranes (Millipore). PVDF membranes were immersed in 5% skim milk, and incubated at 4°C overnight with anti‐PAK4, anti‐RAC2, anti‐cofilin, anti‐E‐cadherin, anti‐GAPDH (Proteintech), and anti‐phospho‐PAK4, anti‐LIMK1, anti‐phospho‐LIMK1, anti‐phospho‐cofilin, anti‐BCL2L1, anti‐PARP1, anti‐N‐cadherin (Cell Signaling Technology) antibodies. HRP‐conjugated secondary antibody (Proteintech) and Enhanced Chemiluminescence Kit (FDbio) were used to detect blots.

### 2.13 Immunohistochemistry (IHC)

Xenograft tumor slides and PCa TMAs were stained by IHC to assess the expression of RAC2/PAK4/BCL2L1. TMAs (HProA150PG01, Xinchao Biotech) comprised 60 cases of PCa samples and paired peritumoral tissues. After dewaxing and rehydrating tissue sections, antigens were retrieved by heating sections in 0.01 mol/L citrate buffer and endogenous peroxidase activities were blocked with 3% hydrogen peroxide. Subsequently, IHC was performed on tumor slides and TMAs with anti‐RAC2, anti‐PAK4 and anti‐BCL2L1 (Proteintech) antibodies at 4°C overnight. IHC staining was achieved by incubating sections with HRP‐conjugated secondary antibody (Proteintech) and DAB development. RAC2/PAK4/BCL2L1 positivity was semiquantitatively analyzed based on the proportion and intensity of the positive cells.

### 2.14 Oncomine database analysis

Oncomine database (https://www.oncomine.org/) was used to analyze mRNA expression pattern of RAC2/BCL2L1 between normal prostate and PCa tissues. The results contained details such as sample size and source, fold of change of mRNA expression, statistical *P* value, and box plot.

### 2.15 Dual‐luciferase reporter assay

The fragments of RAC2/PAK4/BCL2L1 3′‐UTR or PAK4 CDS which contained wild‐type (Wt) or mutant‐type (Mut) binding site of miR‐608 were synthesized as sense and antisense oligos by Sangon. Later they were annealed and inserted into pmirGLO dual‐luciferase vector produced by Promega, between SacⅠ and SalⅠ restriction sites downstream of the firefly luciferase gene. *E. coli* DH5α competent cells (Takara) were used for screening and amplifying the cloned vectors. Correct insertion of Wt or Mut 3′‐UTR/CDS fragments into pmirGLO was confirmed by second‐generation sequencing.

HEK 293T cells were cotransfected with 50 ng dual‐luciferase vector and 50 nmol/L miR‐608 mimic/NC in 48‐well plates. Forty‐eight hours after transfection, relative luciferase activity was measured with E1910 dual‐luciferase reporter system (Promega) and presented as the ratio of the activity of firefly luciferase to that of Renilla luciferase (luc2/Rluc).

### 2.16 RAC2, PAK4, and BCL2L1 rescue experiments

After overnight cultivation, PCa cells were cotransfected with RAC2/PAK4/BCL2L1 siRNA and miR‐608 inhibitor/inhibitor NC and then harvested for the same cell function assays as before. Western blot analyses of RAC2/PAK4/BCL2L1 expression were performed 72 hours after transfection.

### 2.17 Statistical analysis

Research data are presented as mean ± SD. Data normality was determined by Kolmogorov‐Smirnov test. Two‐tailed student's *t *test and one‐way ANOVA test were applied to examine differences between two or more data sets of normal distribution. Differences between paired data sets of abnormal distribution were examined by Wilcoxon sign‐rank test. Statistical analysis was carried out with SPSS 23 software.

## 3 RESULTS

### 3.1 MiR‐608 is low expressed in PCa cell lines and tissues

**Figure 1:**
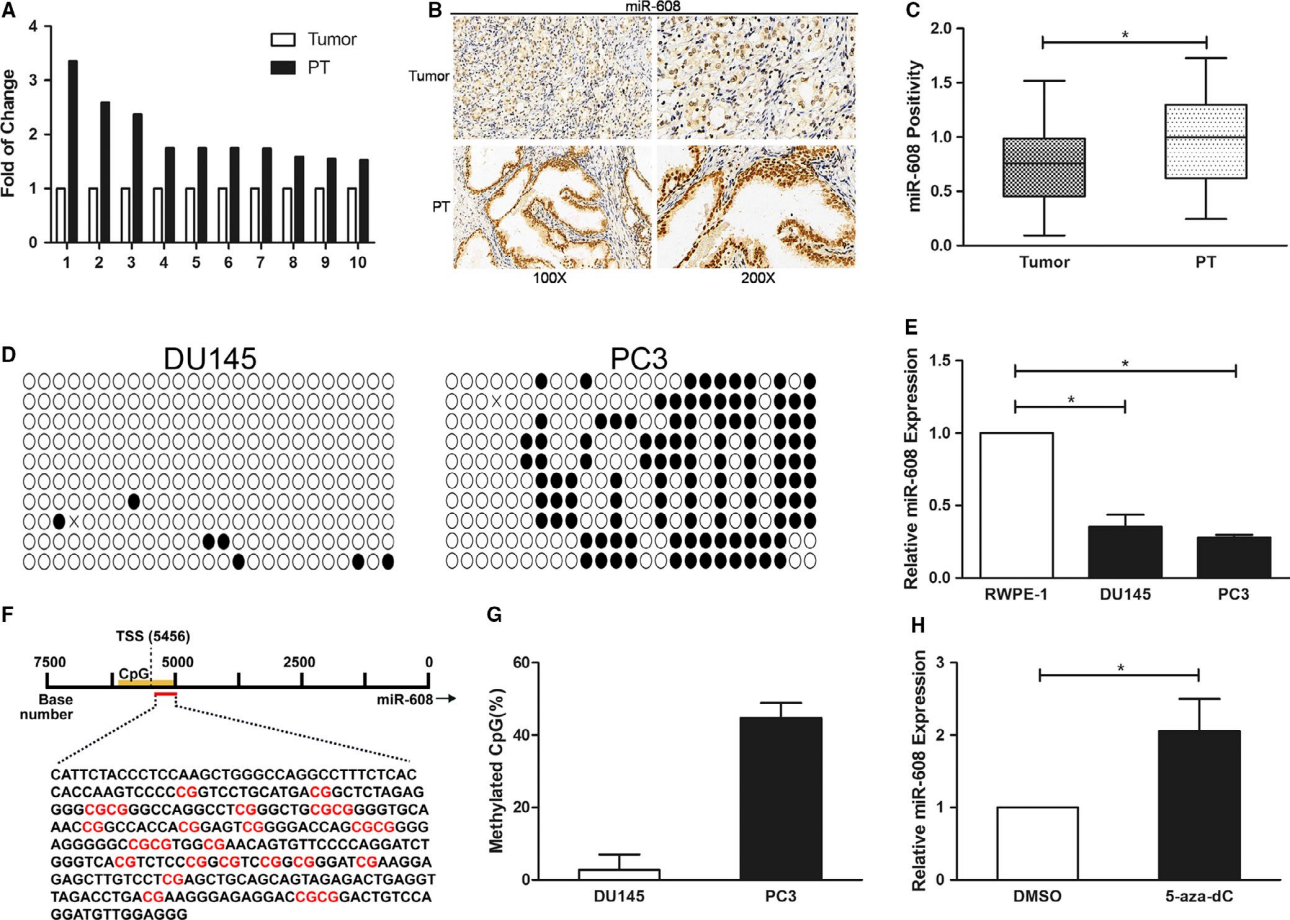
MiR‐608 is low expressed in PCa tissues and is partly correlated with CpG‐island methylation. A, Fold of change of miR‐608 expression in 10 peritumoral tissues (PT) compared to the corresponding PCa tissues. B, Representative results of miR‐608 CISH in PCa TMA. C, Statistical analysis of miR‐608 CISH in PCa TMA. D, The BSP results of the identified CpG island near miR‐608 TSS. The black and white dots represent the methylated and unmethylated CpGs respectively. E, MiR‐608 tailing qRT‐PCR in three prostate cell lines. U6 served as the internal control. F, The DNA sequence of the identified CpG island near miR‐608 TSS with 25 CpGs denoted in red. G, Statistical analysis of the methylation rate of the CpG island in both PCa cell lines. H, The expression of miR‐608 in PC3 cells after 5‐aza‐2′‐deoxycytidine treatment. Error bars represent SD from three independent experiments. *P < .05

To explore miR‐608 expression in PCa and normal prostate cell lines, microRNA tailing qRT‐PCR was adopted to measure miR‐608 expression in DU145 and PC3 PCa cell lines, and in RWPE‐1 prostatic epithelial cell line. In contrast to PCa cell lines, miR‐608 expression increased about threefold in RWPE‐1 (Figure 1E). Subsequently, CISH experiment conducted in PCa TMA validated that miR‐608 expression in PCa tissues remained lower compared to peritumoral tissues, similar to that in PCa and prostatic epithelial cell lines (Figure 1A‐C).

### 3.2 MiR‐608 expression is partly correlated with CpG‐island methylation

MiRNA expression could be epigenetically regulated by CpG‐island methylation, namely methyl groups transferring to the cytosine residues of CpG‐dinucleotide repeats achieved by DNA methyltransferases (DNMT).^13^ Methylated CpG‐island near TSS could silence the corresponding miRNA gene via transcriptive inhibition.^14^, ^15^ Therefore, to explore the mechanisms of miR‐608 downregulation in PCa cell lines and tissues, BSP was employed to investigate the methylation state of CpG‐islands close to miR‐608 TSS.

Online miRStart database (http://mirstart.mbc.nctu.edu.tw/home.php) predicted that miR‐608 TSS was positioned 5456 bps upstream of miR‐608 gene. Next, a CpG‐island was identified near miR‐608 TSS using MethPrimer (http://www.urogene.org/methprimer/) (Figure 1F). BSP revealed that in PC3 cells, the average methylation rate of the CpG‐island was about 40%‐50% (Figure 1D,G), however, there was barely no methylated CpG‐island at the same location in DU145 cells (Figure 1D). Furthermore, to verify that in PC3 cells DNA methylation regulated miR‐608 expression, DNA methylation inhibitor 5‐aza‐2′‐deoxycytidine was utilized to treat PC3 cells, which promoted the expression of miR‐608 in PC3 cells after 72‐hour treatment (Figure 1H). All the results supported the proposition that miR‐608 expression in PC3 cell line was correlated with the CpG‐island methylation close to miR‐608 TSS, while the mechanisms regulating miR‐608 expression in DU145 cell line remain to be probed from other perspectives.

### 3.3 Overexpression of miR‐608 suppresses the proliferation of PCa both in vivo and in vitro

**Figure 2:**
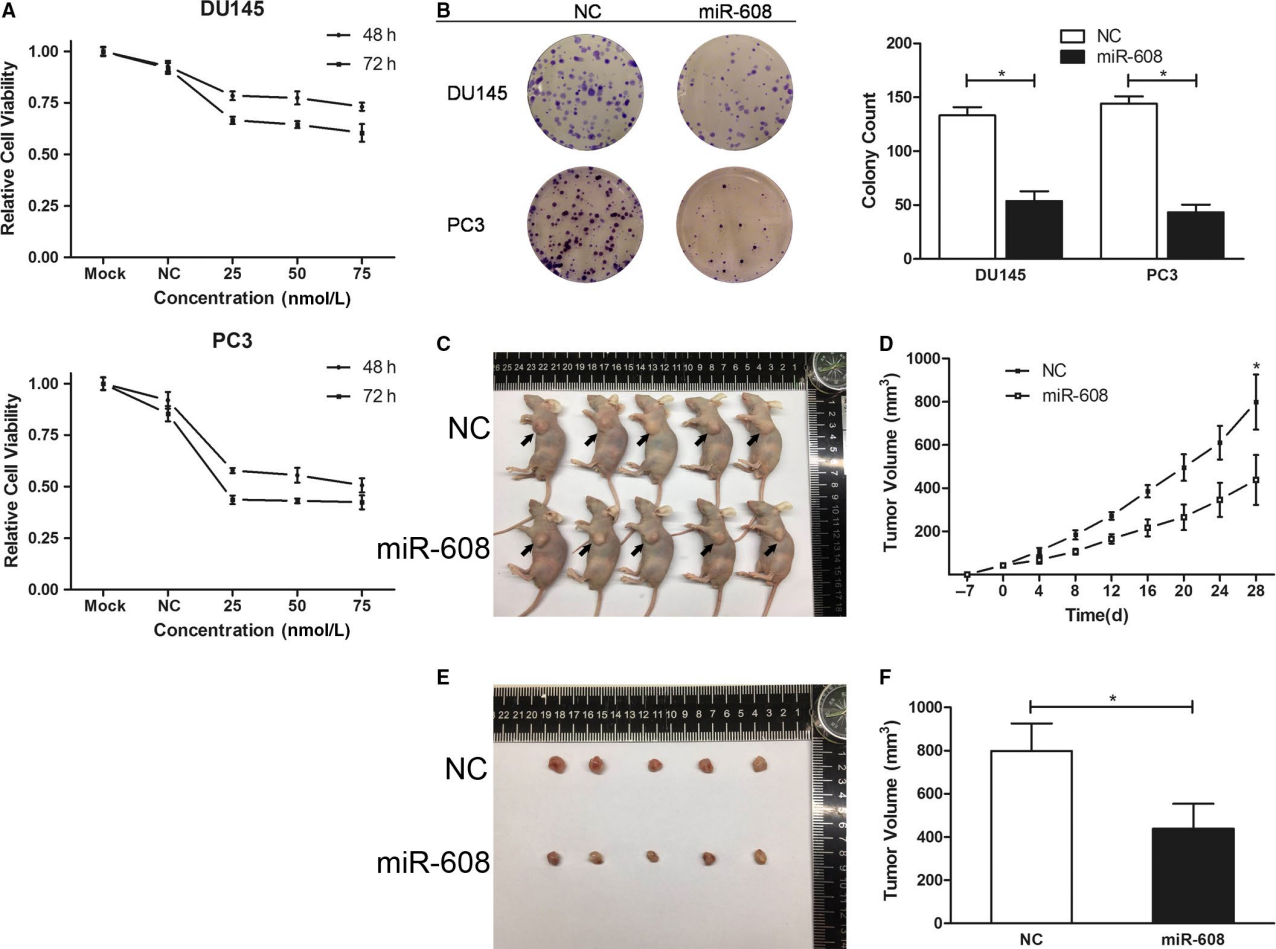
MiR‐608 overexpression suppresses PCa cell proliferation both in vivo and in vitro. A, Representative results of cell viability assay. MiR‐608 mimic of different concentrations was transfected into PCa cells. Error bars represent SD from four replicates. B, Colony formation assay was performed to assess the influence of miR‐608 on PCa cell proliferation. Error bars represent SD from three independent experiments. *P < .05. C, The nude mice sacrificed for the in vivo tumorigenesis experiment. Arrows point to the subcutaneous xenograft tumors. D, The growth curves of the PC3 xenograft tumors of either miR‐608 or NC treated nude mice. Error bars represent SD of the volumes of five xenograft tumors. *P < .05. E, The harvested PC3 xenograft tumors. F, Statistical analysis of the volumes of the harvested xenograft tumors. Error bars represent SD of the volumes of five xenograft tumors. *P < .05

In light of the low background of miR‐608 in PCa, gain‐of‐function strategies were employed to study how miR‐608 influenced the behaviors of PCa cell line DU145 and PC3. As measured by CCK8 assay and colony formation assay, the relative viabilities of DU145 and PC3 cells transfected with miR‐608 mimic decreased considerably (Figure 2A), and their colony‐forming abilities were inhibited as well (Figure 2B). In vivo experiments on nude mice further consolidated the antiproliferative effects of miR‐608 in PCa, in which the growth of PC3 subcutaneous xenograft tumors injected with miR‐608 mimic was significantly slower than those injected with NC (Figure 2C‐F).

### 3.4 Overexpression of miR‐608 induces G2/M arrest of PCa cells

**Figure 3:**
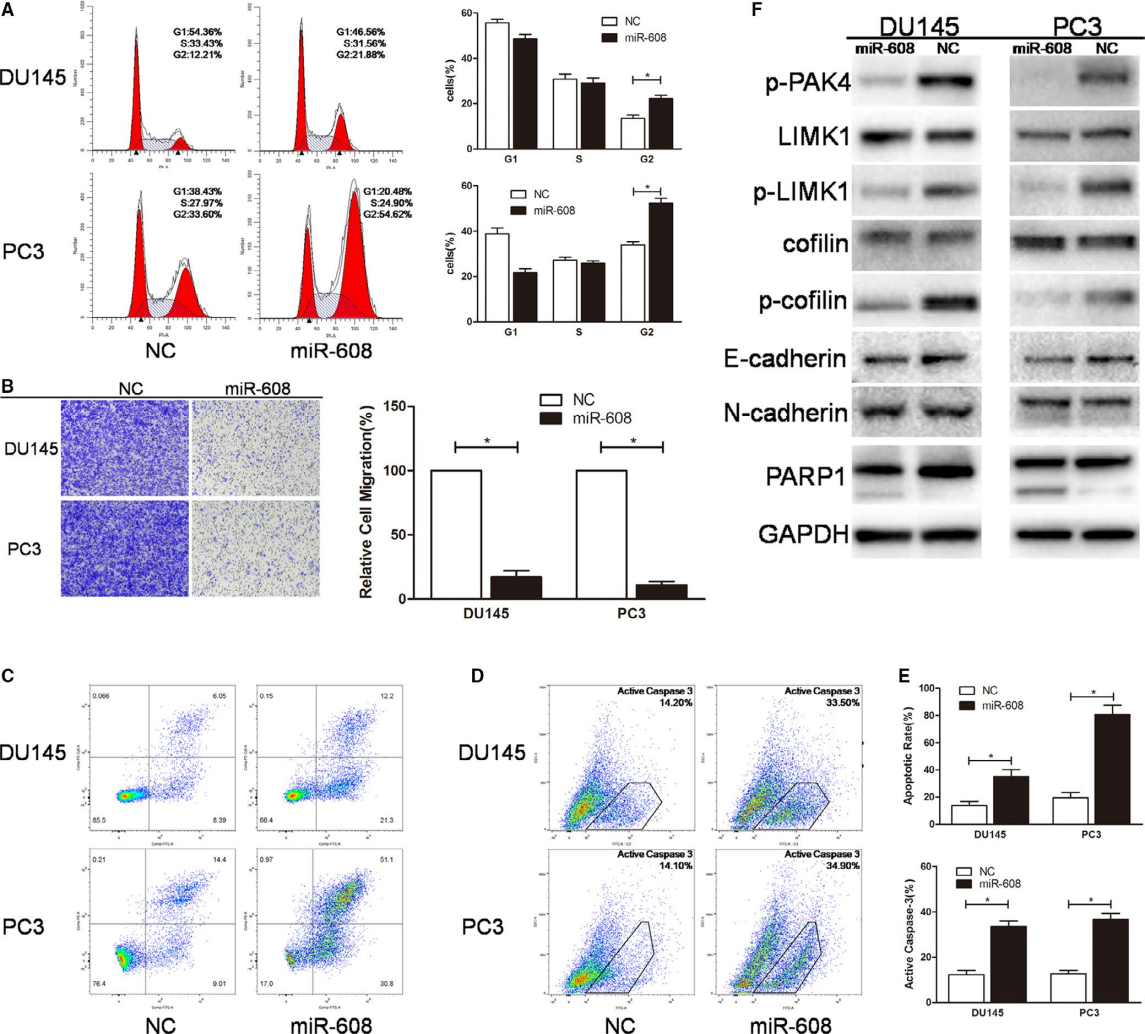
MiR‐608 overexpression induces G2/M arrest, inhibits the migration, and promotes the apoptosis of PCa cells. A, Flow cytometry cell cycle assay. The cell cycle distribution of miR‐608 mimic‐transfected PCa cells was analyzed. B, Transwell migration assay. Cells were observed at 200× magnification. C and D, Flow cytometry apoptosis and active caspase‐3 assay. Apoptotic cells produced by miR‐608 overexpression are in upper right and lower right quadrants. E, Statistical analyses of the apoptosis assay and the active caspase‐3 assay results. F, The expression of miR‐608‐associated proteins after miR‐608 overexpression in PCa cells. Error bars represent SD from three independent experiments. *P < .05

Since cell cycle progression is closely associated with cell proliferation, flow cytometry cell cycles analysis was performed. The results revealed that intracellular overexpression of miR‐608 inhibited G2/M transition of PCa cells, with an appreciably increased proportion of PC3 cells at G2 phase (about 20%) and a moderately increased proportion of DU145 cells at G2 phase (about 8%) (Figure 3A), which indicated delay of mitosis of PCa cells caused by intracellular miR‐608 overexpression.

### 3.5 Upregulation of miR‐608 induces apoptosis of PCa cells

The apoptotic rates of PCa cells after intracellular upregulation of miR‐608 were also determined by flow cytometry apoptosis and active caspase‐3 assays. Upregulation of miR‐608 induced significant apoptosis and caspase‐3 activation in PCa cells compared with NC‐treated cells (Figure 3C‐E), which proved that the antiproliferative mechanisms of miR‐608 in PCa involved apoptosis‐related pathways. Western blot analysis of cleaved PARP1 (poly ADP‐ribose polymerase 1) which was generated by active caspase‐3 further verified the occurrence of caspase‐3‐dependent cell apoptosis in miR‐608 mimic‐transfected PCa cells (Figure 3F).

### 3.6 Inhibition of PCa cell motility by miR‐608 overexpression is independent of EMT‐associated mechanisms

A study on how miR‐608 affected the phenotypes related to PCa cell motility was also carried out by Transwell migration assay. It was evident that intracellular miR‐608 overexpression significantly inhibited the migration of PCa cells, as proved by the sharply decreased transwell migratory rates of miR‐608 mimic‐transfected PCa cells compared with NC‐treated cells (Figure 3B). Interestingly, cadherin expression in PCa cells was not affected by miR‐608 mimic transfection (Figure 3F), which indicated that the antimigratory effects of overexpressed miR‐608 in PCa cells were independent of canonical epithelial‐mesenchymal transition (EMT)‐associated mechanisms.

### 3.7 *MiR‐608 downregulates RAC2 and BCL2L1 by targeting the 3*′*‐UTRs*

**Figure 4:**
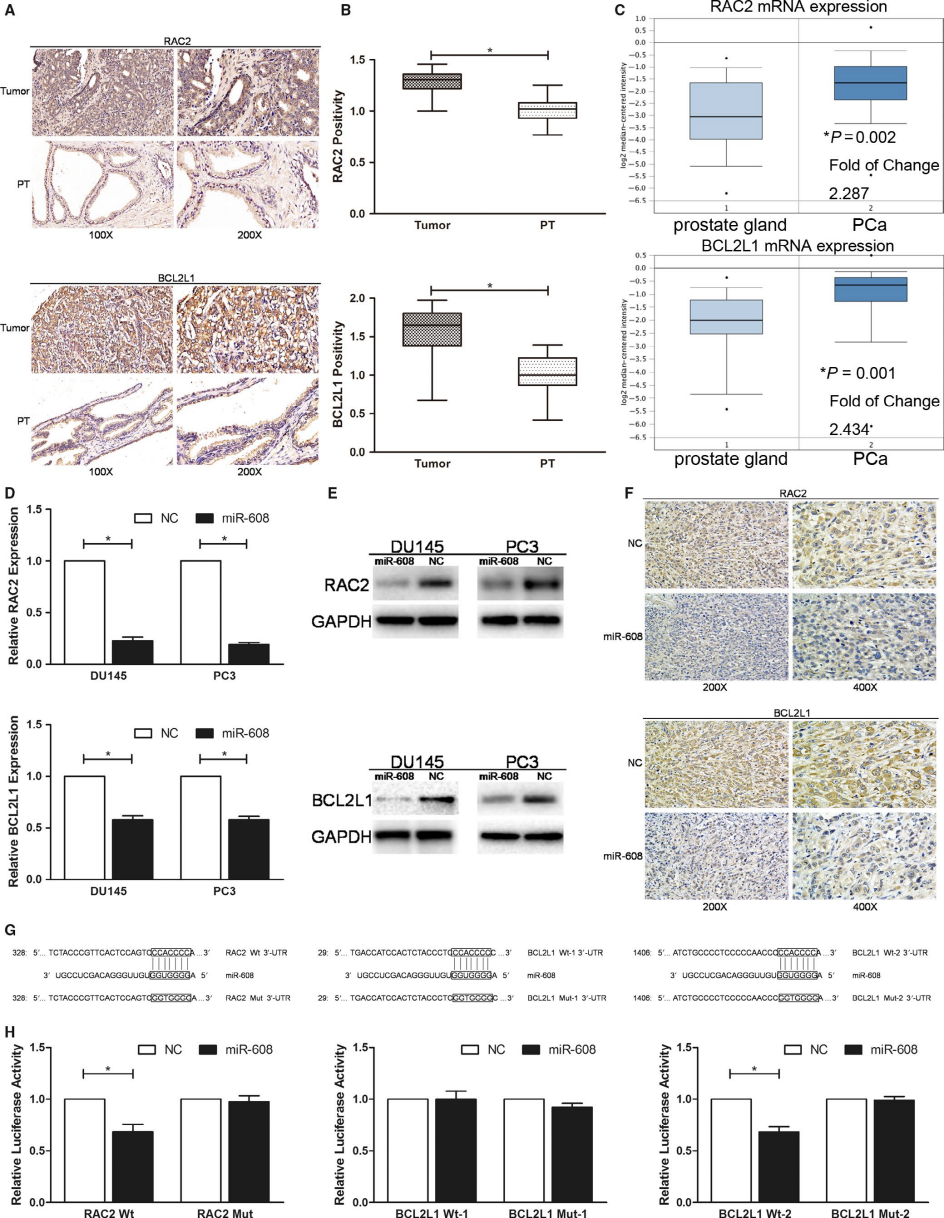
MiR‐608 directly targets RAC2 and BCL2L1 3′‐UTRs. A, Representative results of RAC2/BCL2L1 IHC in PCa TMA which contained 60 pairs of PCa tissues and peritumoral tissues (PT). B, Statistical analysis of RAC2/BCL2L1 IHC in PCa TMA. C, Oncomine analysis of RAC2/BCL2L1 mRNA expression. The results are from Wallace's microarray data sets, which include 69 cases of PCa and 20 cases of normal prostate tissues. D, qRT‐PCR and (E) western blot analysis were applied to determine the expression of RAC2/BCL2L1 after miR‐608 overexpression in PCa cells. F, Representative results of RAC2/BCL2L1 IHC in the sections of the xenograft tumors of either miR‐608 or NC treated nude mice. G, Segments of RAC2/BCL2L1 3′‐UTR containing either the wild‐type (RAC2 Wt/BCL2L1 Wt‐1/BCL2L1 Wt‐2) or the mutant‐type (RAC2 Mut/BCL2L1 Mut‐1/BCL2L1 Mut‐2) miR‐608 binding sites. H, Dual‐luciferase reporter assay. The relative luciferase activity is presented as the ratio of the activity of firefly luciferase to that of Renilla luciferase. Error bars represent SD from three independent experiments. *P < .05

Based on previous cell function assays, microRNA databases were analyzed for the possible targets of miR‐608 in PCa and it was predicted that RAC2 and BCL2L1 were directly targeted by miR‐608. Equally, the in vitro and in vivo studies confirmed that after transfecting PCa cells with miR‐608 mimic, RAC2 and BCL2L1 expression at mRNA and protein levels decreased significantly (Figure 4D‐F). Meanwhile, the proteins downstream of RAC2 were downregulated as well (Figure 3F). Besides, analyses of RAC2 and BCL2L1 expression patterns using TMA IHC and oncomine database analysis indicated that they were upregulated in PCa tissues compared with peritumoral tissues (Figure 4A‐C).

Dual‐luciferase reporter assays confirmed that RAC2 and BCL2L1 were directly targeted by miR‐608 at the 3′‐UTRs. The segment of RAC2 3′‐UTR containing the wild‐type (RAC2 Wt) or the mutant‐type (RAC2 Mut) miR‐608 binding site was incorporated into pmirGLO dual‐luciferase vector (sequences shown in Figure 4G and Table 1) where the segment was located downstream of the firefly luciferase gene. Similarly, two different segments of BCL2L1 3′‐UTR containing either the wild‐type (BCL2L1 Wt‐1 and BCL2L1 Wt‐2) or the corresponding mutant‐type (BCL2L1 Mut‐1 and BCL2L1 Mut‐2) binding sites were inserted into pmirGLO in the same way (sequences shown in Figure 4G and Table 1).

Dual‐luciferase reporter assay was conducted separately for each 3′‐UTR construct. Downstream of the firefly luciferase gene, targeting of the 3′‐UTR segment by miR‐608 downregulated the expression of the firefly luciferase (luc2) while the expression of the Renilla luciferase (Rluc) was unchanged, which decreased the relative luciferase activity. The results demonstrated that compared with NC, miR‐608 downregulated the relative luciferase activities of RAC2 Wt/BCL2L1 Wt‐2 vector‐transfected HEK 293T cells, whereas miR‐608 did not significantly change the relative luciferase activities of HEK 293T cells which were transfected with RAC2 Mut/BCL2L1 Mut‐2 or BCL2L1 Wt‐1/BCL2L1 Mut‐1 vectors. This supported that RAC2 and BCL2L1 were direct targets of miR‐608 (Figure 4H).

### 3.8 MiR‐608 downregulates PAK4 by targeting the CDS

**Figure 5:**
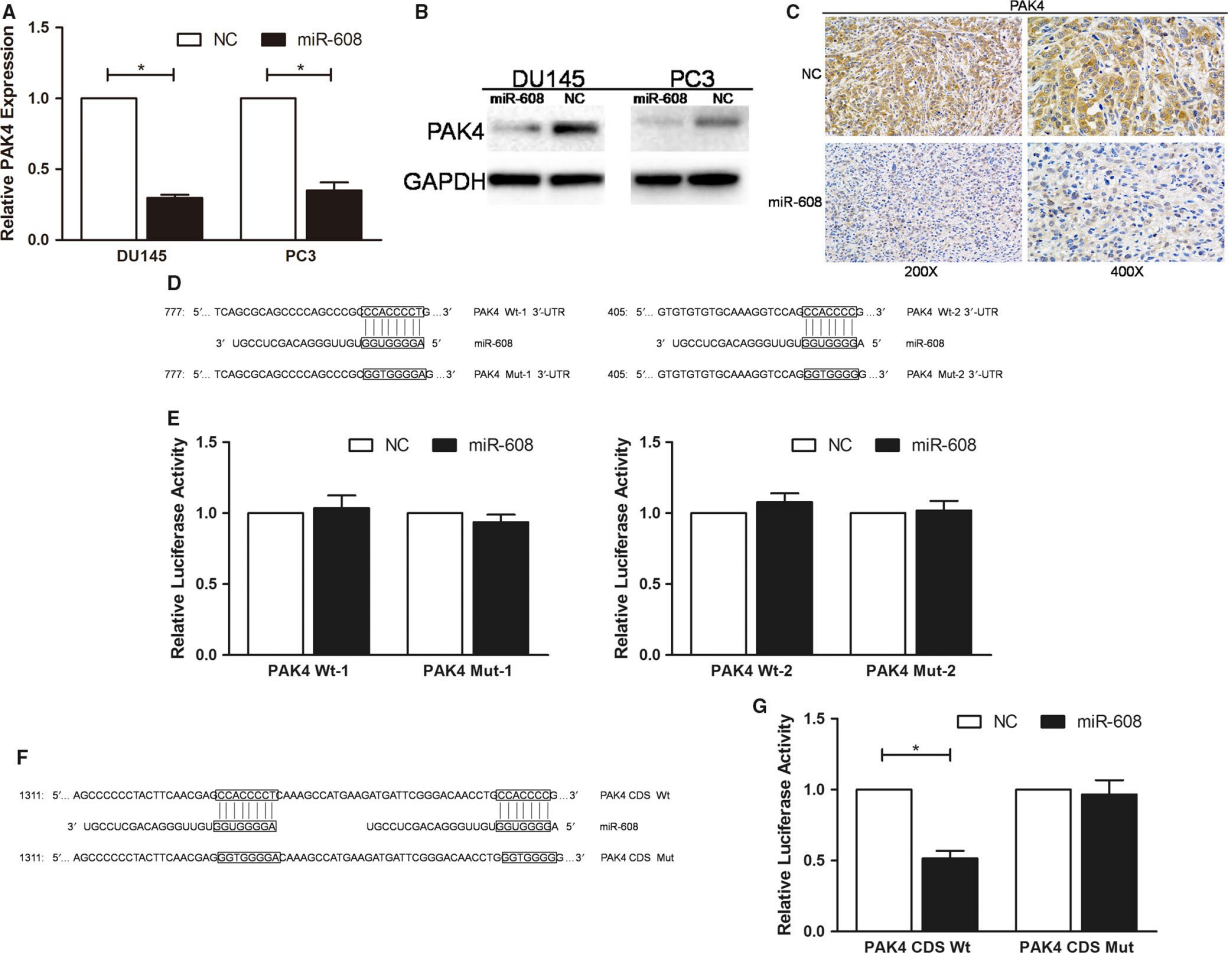
MiR‐608 targets the PAK4 CDS. A, qRT‐PCR and (B) western blot analysis were applied to determine the expression of PAK4 after miR‐608 overexpression in PCa cells. C, Representative results of PAK4 IHC in the sections of the xenograft tumors of either miR‐608 or NC treated nude mice. D, Segments of PAK4 3′‐UTR containing either the wild‐type (PAK4 Wt‐1 and PAK4 Wt‐2) or the mutant‐type (PAK4 Mut‐1 and PAK4 Mut‐2) miR‐608 binding sites. E, Dual‐luciferase reporter assay. MiR‐608 mimic had no effects on the relative luciferase activities of PAK4 3′‐UTR Wt vector‐transfected HEK 293T cells. F, Segments of PAK4 CDS containing either the wild‐type (PAK4 CDS Wt) or the mutant‐type (PAK4 CDS Mut) miR‐608 binding sites. G, Dual‐luciferase reporter assay. The relative luciferase activity is presented as the ratio of the activity of firefly luciferase to that of Renilla luciferase. Error bars represent SD from three independent experiments. *P < .05

**Figure 6:**
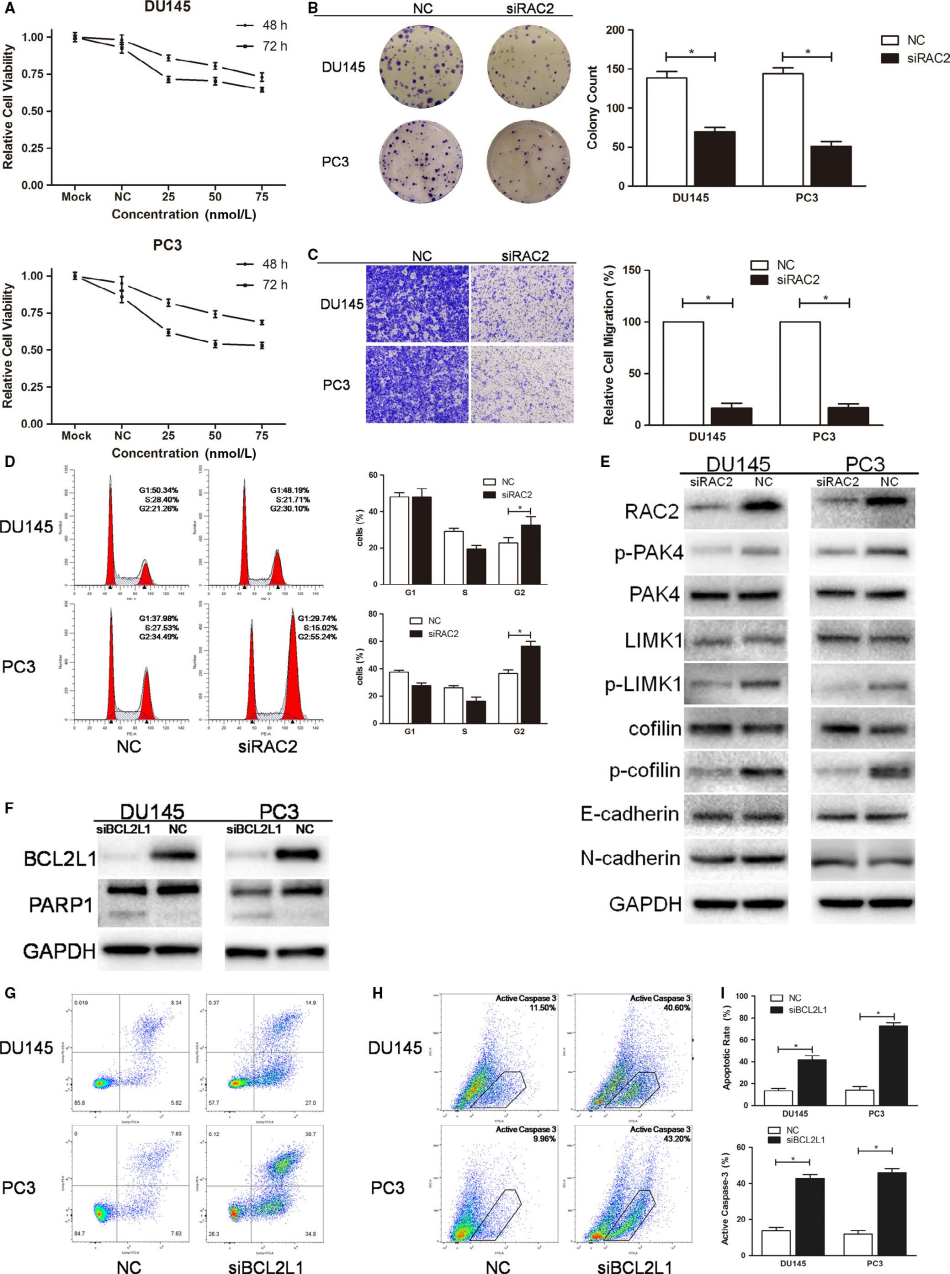
Knocking down RAC2/BCL2L1 with RAC2/BCL2L1 siRNA pool mimics the effects of miR‐608 in PCa cells. A, Representative results of cell viability assay. RAC2 siRNA of different concentrations was transfected into PCa cells. Error bars represent SD from four replicates. B, Colony formation assay was performed to assess the influence of RAC2 siRNA on PCa cell proliferation. C, Transwell migration assay. Cells were observed at 200× magnification. D, Flow cytometry cell cycle assay. The cell cycle distribution of RAC2 siRNA‐transfected PCa cells was analyzed. E and F, Western blot analysis. RAC2/BCL2L1 siRNA pool knocked down RAC2/BCL2L1 and the downstream proteins in PCa cells. G and H, Flow cytometry apoptosis and active caspase‐3 assay. Apoptotic cells produced by BCL2L1 knock down are in upper right and lower right quadrants. I, Statistical analyses of the apoptosis assay and the active caspase‐3 assay results. Error bars represent SD from three independent experiments. *P < .05

As both in vitro and in vivo, miR‐608 downregulated PAK4 at mRNA and protein levels in PCa cells (Figure 5A‐C), it was postulated that miR‐608 might also target PAK4 in PCa. However, vectors inserted with any of the 3′‐UTR of PAK4 mRNA (PAK4 Wt‐1 and Wt‐2) which contained miR‐608 binding site were all negative for the dual‐luciferase reporter assays (Figure 5D,E), indicating that the 3′‐UTR of PAK4 mRNA was not targeted by miR‐608. According to the western blot results, knocking down RAC2 with siRNA pool in PCa cells only reduced the expression of phosphorylated PAK4, but not unphosphorylated PAK4 (Figure 6E), which suggested that the downregulation of PAK4 by miR‐608 was not attributed to its downregulation of RAC2. Moreover, in the CDS of PAK4 mRNA, two sequences were discovered which were 31 nucleotides apart and completely complementary to the seed region of miR‐608 (Figure 5F).

All the experimental and theoretical facts pointed to the possibility that miR‐608 might target PAK4 by interacting with the CDS of PAK4 mRNA instead of the canonical 3′‐UTR. Considering that dual‐luciferase reporter assay is also a widely accepted method for studying miRNA‐CDS interactions,^6^, ^16^, ^17^, ^18^ the segment of PAK4 CDS (named as PAK4 CDS Wt) which contained the two sequences complementary to miR‐608 seed region was inserted into pmirGLO as conducted in the last part. The vector with the corresponding mutant sequences was also constructed (named as PAK4 CDS Mut) (Figure 5F). The results showed that compared with NC, miR‐608 dramatically downregulated the relative luciferase activities of PAK4 CDS Wt vector‐transfected HEK 293T cells, but that difference was not observed in PAK4 CDS Mut vector‐transfected HEK 293T cells. This corroborated that miR‐608 targeted the CDS of PAK4 (Figure 5G).

### 3.9 Knocking down RAC2, PAK4, or BCL2L1 mimics the effects of miR‐608 in PCa which could be attenuated by miR‐608 inhibitor

**Figure 7:**
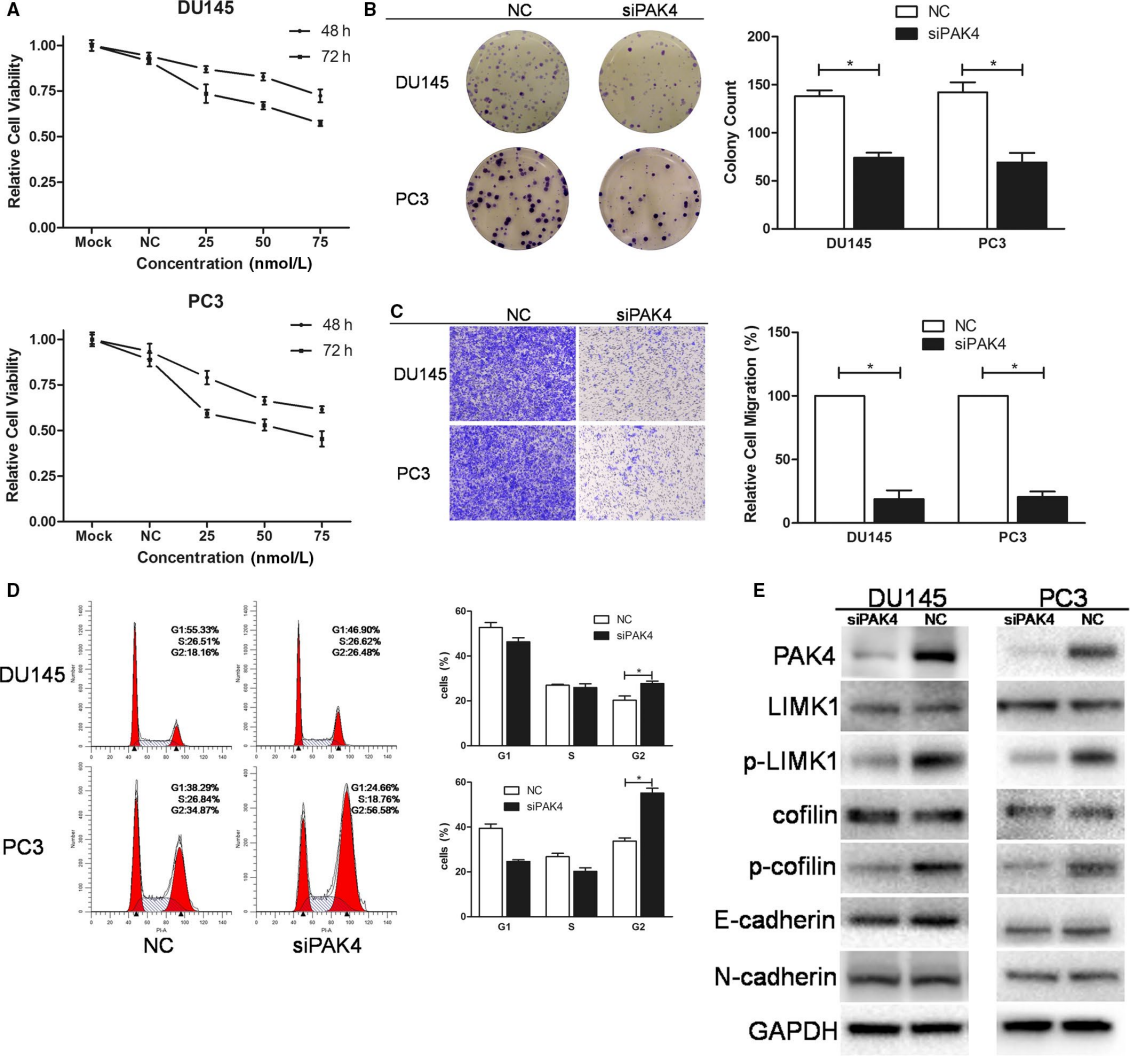
Knocking down PAK4 with PAK4 siRNA pool mimics the effects of miR‐608 in PCa cells. A, Representative results of cell viability assay. PAK4 siRNA of different concentrations was transfected into PCa cells. Error bars represent SD from four replicates. B, Colony formation assay was performed to assess the influence of PAK4 siRNA on PCa cell proliferation. C, Transwell migration assay. Cells were observed at 200× magnification. D, Flow cytometry cell cycle assay. The cell cycle distribution of PAK4 siRNA‐transfected PCa cells was analyzed. E, Western blot analysis. PAK4 siRNA pool knocked down PAK4 and the downstream proteins in PCa cells. Error bars represent SD from three independent experiments. *P < .05

Since RAC2, PAK4, and BCL2L1 were validated to be targeted by miR‐608, cell function assays using siRNAs of RAC2/PAK4/BCL2L1, and RAC2/PAK4/BCL2L1 rescue experiments were conducted to further elucidate the association between miR‐608 and RAC2/PAK4/BCL2L1 in PCa. In order to diminish off‐target effects of RNA interference, two different siRNA sequences were designed and synthesized for each target mRNA, and were mixed as a siRNA pool for the protein knocking down and the related cell function assays (sequences shown in Table 1). The results demonstrated that knocking down RAC2/PAK4 with siRNA pool reproduced the antiproliferative, mitosis‐obstructive, and antimigratory effects of miR‐608 overexpression in PCa cells (Figures 6 and 7), and knocking down BCL2L1 with siRNA pool reproduced the proapoptotic effect of miR‐608, which also involved caspase‐3 activation (Figure 6F‐I). Besides, similar to miR‐608, cadherin expression in PCa cells was not affected by RAC2/PAK4 siRNA transfection (Figures 6E and 7E), which further confirmed that EMT‐associated mechanisms were not implicated in the miR‐608‐induced inhibition of PCa cell motility.

**Figure 8:**
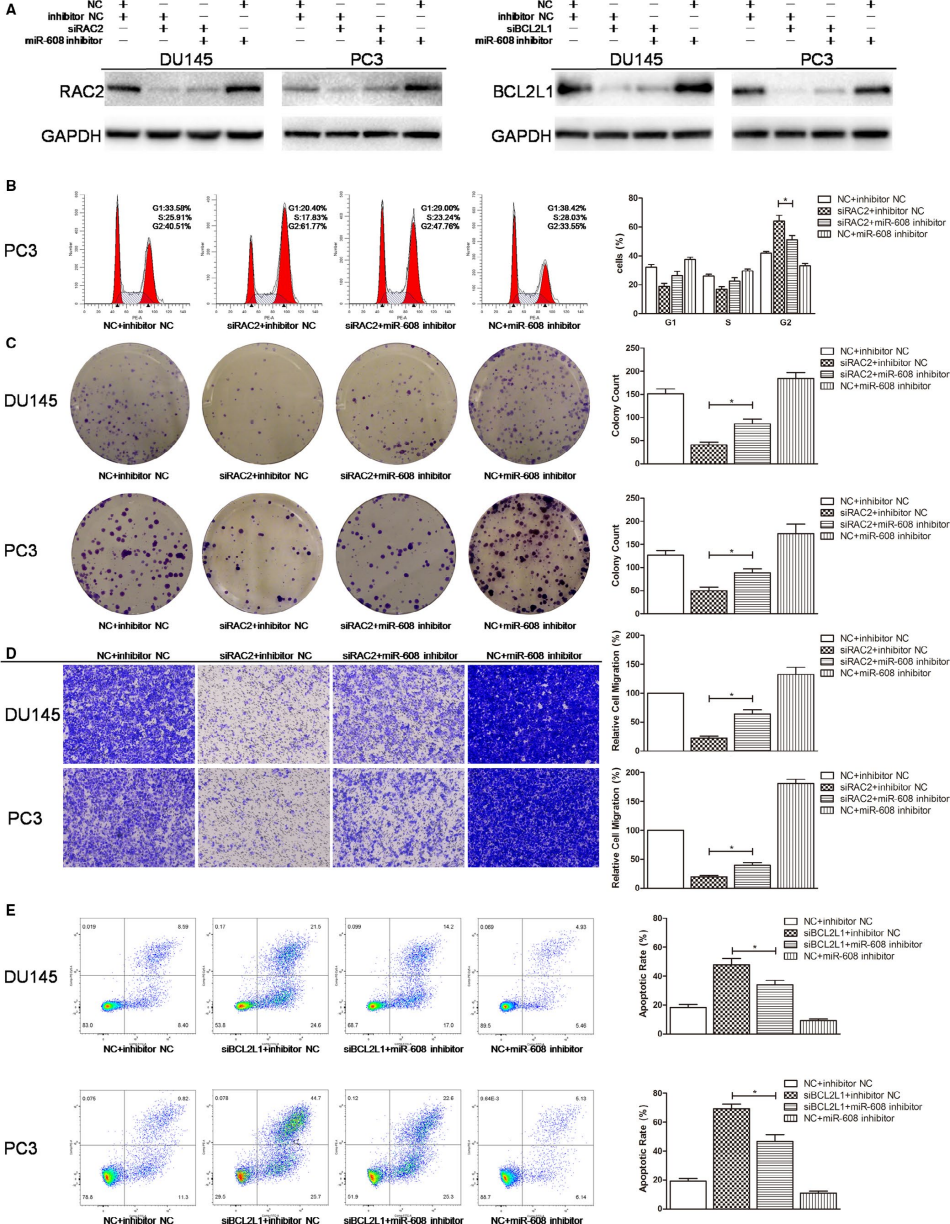
RAC2/BCL2L1 rescue experiments. A, Western blot analysis. MiR‐608 inhibitor re‐elevated the expression of RAC2/BCL2L1 in RAC2/BCL2L1 knocked‐down PCa cells. B, Flow cytometry cell cycle assay. MiR‐608 inhibitor abolished the G2/M arrest caused by RAC2 knock down in PCa cells. C, Colony formation assay. MiR‐608 inhibitor partially recovered the proliferation of RAC2 knocked‐down PCa cells. D, Transwell migration assay. MiR‐608 inhibitor restimulated the migration of RAC2 knocked‐down PCa cells. E, Flow cytometry apoptosis assay. MiR‐608 inhibitor significantly reduced the apoptosis caused by BCL2L1 knock down in PCa cells. Error bars represent SD from three independent experiments. *P < .05

**Figure 9:**
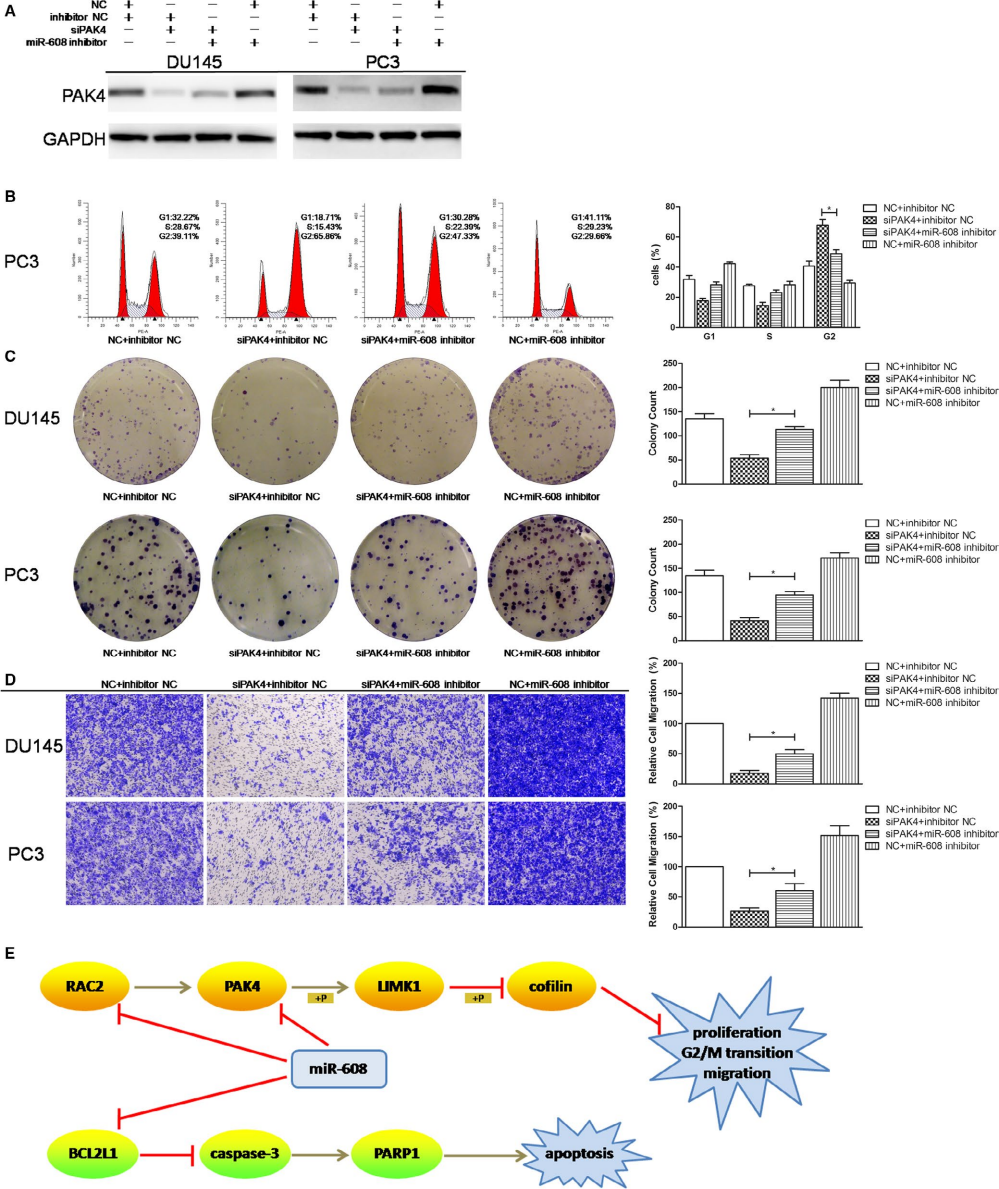
PAK4 rescue experiments. A, Western blot analysis. MiR‐608 inhibitor re‐elevated the expression of PAK4 in PAK4 knocked‐down PCa cells. B, Flow cytometry cell cycle assay. MiR‐608 inhibitor abolished the G2/M arrest caused by PAK4 knock down in PCa cells. C, Colony formation assay. MiR‐608 inhibitor partially recovered the proliferation of PAK4 knocked‐down PCa cells. D, Transwell migration assay. MiR‐608 inhibitor restimulated the migration of PAK4 knocked‐down PCa cells. E, Summary of the mechanisms of miR‐608 in PCa. Error bars represent SD from three independent experiments. *P < .05

In RAC2/PAK4/BCL2L1 rescue experiments, cotransfection of miR‐608 inhibitor re‐elevated the expression of RAC2, PAK4, and BCL2L1 in RAC2/PAK4/BCL2L1 knocked‐down PCa cells (Figures 8A and 9A). Furthermore, miR‐608 inhibitor cotransfection significantly increased the colony formation rates and the transwell migratory rates of RAC2/PAK4 siRNA‐transfected PCa cells (Figures 8C,D and 9C,D), and significantly decreased the apoptotic rates of BCL2L1 siRNA‐transfected PCa cells (Figure 8E). At the same time, miR‐608 inhibitor abrogated the G2/M arrest induced by RAC2/PAK4 knock down in PC3 cells, characterized by a reduced proportion of PC3 cells at G2 phase (Figures 8B and 9B). Finally, NC+miR‐608 inhibitor‐transfected PCa cells exhibited the highest level of RAC2/PAK4/BCL2L1 expression, the highest proliferative and migratory capabilities, and the lowest apoptotic rates (Figures 8 and 9). All of these findings further substantiated the targeting of RAC2/PAK4/BCL2L1 by miR‐608. The functions of miR‐608 in PCa and the relevant mechanisms were summarized in Figure 9E.

## 4 DISCUSSION

MiR‐608 is a nonconserved miRNA, residing in the intron of human SEMA4G (semaphorin 4G) gene.^8^ This is the first research to systematically explore the tumor‐suppressive roles of miR‐608 in PCa. Meanwhile, RAC2, a GTPase previously considered to specifically exist and function in cells of hematopoietic origin, such as blood cells, immune cells, and bone marrow,^19^, ^20^ was detected in PCa and confirmed to be targeted by miR‐608 in PCa. Also, we demonstrated the upregulation of RAC2 in PCa in comparison with peritumoral tissues. Knocking down RAC2 by either RAC2 siRNA or miR‐608 mimic suppressed the proliferation, induced G2/M arrest, and inhibited the migration of PCa cells.

RAC2 belongs to RAC family (Ras‐related C3 botulinum toxin substrate), which is a subgroup of Rho (Ras homolog GTPase) family. All Rho GTPases are capable of interacting with effector proteins and triggering diverse biological responses when bound with GTP.^21^, ^22^ All RACs stabilize actin filaments, and thus are closely associated with organization of cytoskeleton, construction of cell pseudopodia, and cell migration.^23^, ^24^, ^25^ RACs function through conformationally activating PAKs (p21‐activated kinase) and promoting their auto‐phosphorylation, and the phosphorylation status of PAK is an indicator of PAK kinase activity.^26^, ^27^ In particular, PAK4 is activated by phosphorylation at Ser^474^.^28^, ^29^ We found that knocking down RAC2 decreased PAK4 phosphorylation, and knocking down PAK4 generated similar effects on PCa cell behaviors to RAC2 knock down. Therefore, RAC2 and PAK4 are colocalized in one signaling pathway and miR‐608 could target both of them. In human, PAK4 was upregulated in breast cancer, ovarian cancer, and prostate cancer, where PAK4 exerts tumorigenic effects by enhancing cell proliferation and cell migration.^28^, ^30^, ^31^

The biological effects of activated PAKs are fulfilled via LIMKs (LIM kinase) and cofilin. LIMKs are activated through phosphorylation of their conserved threonine residues. For instance, PAKs phosphorylate Thr^508^ of LIMK1 and ROCKs phosphorylate Thr^505^ of LIMK2.^32^, ^33^ Besides, in vitro experiments showed that PAK4 preferentially phosphorylated LIMK1 over LIMK2.^34^, ^35^ Cofilin, an actin depolymerizing factor (ADF), could expedite actin filament turnover—cofilin cleaves actin filaments, thus generating free terminals where new filaments grow and supplying actin monomers for actin filament elongation.^36^, ^37^ LIMK1 inactivates cofilin by phosphorylating its Ser^3^ residue, which suppresses severance of actin filaments and stabilizes them.^24^, ^25^

Since cofilin‐induced turnover of filamentous actin is necessary for actin polymerization and pseudopodia formation, LIMK1 was first recognized as a cell migration inhibitor because it inactivates cofilin. For example, overexpression of LIMK1 kinase domain abolished pseudopodia extension and cell migration of rat metastatic mammary adenocarcinoma cell MTLn3.^38^ In mouse myoblast C2C12 cell, activation of PAK4 or LIMK1 decreased actin severance, caused aberrant polymerization of actin filaments and reduced pseudopodia protrusion, which was reversed by expressing constitutively activated cofilin.^34^

However, according to the findings of this research, inactivation of LIMK1 by intracellular introduction of miR‐608 mimic or RAC2/PAK4 siRNA significantly impeded PCa cell motility, indicating LIMK1 as a cell migration promoter in PCa. In fact, inconsistencies with reference to the effects of LIMK1 on cell pseudopodia formation and cell motility have been reported in the past. Nishita et al showed that stromal cell‐derived factor 1 (SDF‐1) stimulated Jurkat T‐cell chemotaxis by activating RAC1/LIMK1/cofilin pathway, and suppression of LIMK1 greatly inhibited pseudopodia extension and T‐cell movement.^39^ LIMK1 also enhanced pseudopodia formation and cell migration in PCa^40^ and breast cancer,^41^ which were similar to the findings of this study. One possible explanation for this inconsistency is that aside from dynamic turnover of actin filaments, their optimal stabilization might also be necessary for pseudopodia maintenance and cell movement. The question on how LIMK1 activation and cofilin inactivation contribute to PCa cell migration requires more research in the future.

Aside from pseudopodia formation and cell migration, PAK4, LIMK1, and cofilin are also closely associated with cell mitosis. It was reported that during mitosis, PAK4 and LIMK1 were localized to the centrosome,^42^, ^43^ and cofilin was accumulated in the contractile ring, which suggested their roles in mitotic cytokinesis.^44^ Periodic changes of LIMK1 and cofilin activities in the process of cell division were observed as well, as p‐LIMK1 and p‐cofilin expression increased at early stages of mitosis, and decreased at later stages.^45^ More importantly, requirements of PAK4, LIMK1, and cofilin phosphorylation for correct orientation and positioning of mitotic spindles have been elucidated.^46^, ^47^ All the evidences explained the G2/M arrest of PCa cells caused by intracellular miR‐608 overexpression or RAC2/PAK4 knock down. Interestingly, knocking down LIMK1 did not change the expression of CDK1 (cyclin‐dependent kinase 1), AURKA (aurora kinase A), or PLK1 (polo‐like kinase 1),^48^ indicating that the mitosis‐promotive effects of PAK4/LIMK1/cofilin signaling were independent of the canonical G2/M transition‐associated proteins.

BCL2L1 (B‐cell lymphoma 2 like 1, also known as BCL‐x) belongs to BCL2 family and is an important inhibitor of mitochondria‐related cell apoptosis. Upon exogenous or endogenous cellular stress, Bax and Bak that belong to the BCL2 family are activated through conformational changes and oligomerize on the mitochondrial outer membrane, which facilitates its opening and promotes the release of mitochondrial cytochrome C into cytosol. Next, caspase‐3 is activated by cytochrome C and initiates serial downstream apoptotic responses. To prevent the opening of the mitochondrial outer membrane, BCL2L1 binds to Bax and Bak and inhibits their oligomerization, and thus functions as an antiapoptotic factor.^49^ We demonstrated the upregulation of BCL2L1 in PCa in comparison with peritumoral tissues. MiR‐608 overexpression induced significant apoptosis in PCa by directly targeting BCL2L1 through BCL2L1/caspase‐3 signaling pathway.

It was noticed that despite the same origin of prostate cancer, DU145 and PC3 cell lines exhibited different phenotypes regarding their epigenetic modification patterns, as well as functional changes induced by intracellular overexpressed miR‐608. While miR‐608 transfection drastically blocked PC3 cells at G2 phase, it only slightly elevated the proportion of DU145 cells at G2 phase. In comparison with the moderate methylation rate of the CpG‐island close to miR‐608 TSS in PC3 cell line, there was hardly any methylated CpG‐island at the same location in DU145 cell line. Given the idiosyncrasies of different cell lines, this conveys the significance of studying specific miRNAs in the contexts of specific physiological or pathological situations.

One thing that was also noteworthy was the differential expression of miR‐608/RAC2/BCL2L1 in PCa cells and tissues in comparison with prostatic epithelial cells and peritumoral tissues, which provided their diagnostic potentials in PCa. However, being commercialized products, the TMAs had no information about the survival duration, hence the absent analysis of the correlation between miR‐608/RAC2/BCL2L1 expression and PCa patient prognosis is a limitation of this study. The analyses of the prognostic values of miR‐608, RAC2 and BCL2L1 in PCa have to be based on more detailed information and larger sample size in the future.

In conclusion, miR‐608 is low expressed in PCa cell lines and tissues, and suppresses PCa progression. In PCa, it inhibits cell proliferation, induces cell G2/M arrest, and inhibits cell migration by targeting the 3′‐UTRs of RAC2 and BCL2L1, and the CDS of PAK4. The inhibitory effects of miR‐608 in PCa are dependent on RAC2/PAK4/LIMK1/cofilin and BCL2L1/caspase‐3 signaling pathways.

## CONFLICT OF INTEREST

The authors declare no conflict of interest.

## AUTHOR CONTRIBUTIONS

Xu Zhang, Jiajie Fang, Shiming Chen, and Weiyu Wang were involved in experiment design and conduction. Xu Zhang, Jiajie Fang, Shiming Chen, Weiyu Wang, and Shuai Meng were involved in data analysis. Xu Zhang and Jiajie Fang were involved in writing. Shuai Meng and Ben Liu were involved in manuscript review and edit. Ben Liu was involved in Supervision.
